# Reply to Beechar et al. “Donor-Derived Infections: A Journey From Evidence to Policy”

**DOI:** 10.1093/ofid/ofae671

**Published:** 2024-11-12

**Authors:** Carl Boodman, Oscar Fernandez Garcia, Dima Kabbani, Armelle Perez Cortes Villalobos, Johan van Griensven, Karen Doucette

**Affiliations:** Department of Internal Medicine, Division of Infectious Diseases, University of Manitoba, Winnipeg, Manitoba, Canada; Unit of Neglected Tropical Diseases, Institute of Tropical Medicine, Antwerp, Belgium; Department of Infectious Disease, Faculty of Medicine & Dentistry Medicine, University of Alberta, Edmonton, Alberta, Canada; Department of Infectious Disease, Faculty of Medicine & Dentistry Medicine, University of Alberta, Edmonton, Alberta, Canada; Department of Internal Medicine, Division of Infectious Diseases, University of Manitoba, Winnipeg, Manitoba, Canada; Unit of Neglected Tropical Diseases, Institute of Tropical Medicine, Antwerp, Belgium; Department of Infectious Disease, Faculty of Medicine & Dentistry Medicine, University of Alberta, Edmonton, Alberta, Canada

To the  Editor—We thank Beechar et al for their response [1] to our article on donor-derived *Bartonella quintana* infection in solid organ transplants [2]. Beechar and colleagues highlight important points regarding the necessity of evaluating the consequences of screening high-risk solid organ transplant donors and recipients for *Bartonella quintana* infection. We wholeheartedly agree with the need for more data and clearly articulated this opinion in our article, suggesting that prospective studies occur in collaboration with organ procurement organizations.

In our article, we described the novel occurrence of donor-derived *B quintana* infection in multiple jurisdictions over the last 2 years [2]. We proposed a system to screen and monitor individuals at elevated risk of donor-derived *B quintana*, extrapolating from the published literature and our clinical experience managing cases of *B quintana* disease. We articulated the limitations of diagnostic tests for *B quintana* and described how these problems may be amplified in the transplantation context, outlining the need for a multipronged approach. As with all emerging health phenomena, robust data are limited at the beginning, especially for neglected infections linked to poverty, such as *B quintana.* While Beechar et al warn of unforeseen consequences for organ utilization [1], we explicitly articulated that *B quintana* screening should not interrupt organ transplantation; it is expected that organs will be transplanted before *B quintana* test results. We emphasized that even if a positive test result happens to be identified before organ procurement, it should not delay transplantation, as *B quintana* is treatable. While all diagnostic tests may be associated with false-positive results, we outlined the epidemiologic risk factors for *B quintana* and suggested that *B quintana* testing be reserved for individuals with a high pretest probability of infection. Our proposed combination of different diagnostic tests and epidemiologic risk factors was an attempt to mitigate false-positivity.

In a more detailed account of the cluster in Alberta, Canada, Kabbani et al [3] describe 6 cases of donor-derived *B quintana* disease originating from 11 seropositive donors. This exemplifies the role of donor serology in identifying recipients at elevated risk of donor-derived infection. Beyond the initial cluster, more cases of donor-derived *B quintana* have since been identified in Alberta [4]. While the Albertan cases were predominantly cutaneous bacillary angiomatosis, the US cluster encompassed severe manifestations of donor-derived *B quintana* infection, including a case of infective endocarditis [5]. This endocarditis case was minimally symptomatic and detected only through active screening of organ transplant recipients after severe *B quintana* disease was diagnosed in another organ transplant recipient from the same donor [5]. *B quintana* infective endocarditis is a disease with fatality rate of approximately 10% despite surgical and antimicrobial treatment, and the mortality rate may be higher among individuals with compromised immune systems [6]. Furthermore, cases of *B quintana* endocarditis are often diagnosed many months after symptoms onset, further emphasizing the need to identify those at greatest risk early and prevent unnecessary disease and death [6]. Cost-effectiveness analyses, as suggested by Beechar and colleagues [1], should thus incorporate the cost savings of preventing *B quintana* endocarditis, a costly disease that often necessitates cardiovascular surgery, intensive care, and prolonged hospital admission to treat.

Guideline recommendations should preferentially be based on robust studies. However, when a new phenomenon is described and data are lacking, guidelines may be used to catalyze data acquisition and improve the evidence base, while remaining transparent about the rationale for any recommendation. Thus, we believe that the opinion expressed by Beechar et al may be limited by circular reasoning; one cannot build up the evidence base for an emerging disease without a framework for testing. When distilled to its essence, our article was an appeal for more information, presented as an algorithm to facilitate data collection. *B quintana* is a disease of poverty, with little to no research funding and few research laboratories globally. Despite its public health relevance, global distribution, and ability to cause fatal disease, the infection is not notifiable in any jurisdiction, and thus there is no centralized information on its epidemiology [7]. Beechar and colleagues appropriately highlight the Organ Procurement Transplant Network (OPTN)'s first policy step to guide screening for emerging pathogens in donors: determining the magnitude of the problem [8]. We cannot agree more. However, one cannot determine the magnitude of donor-derived *B quintana* infection without a diagnostic algorithm. If we wait for high-quality studies on neglected infections before proposing a framework for testing high-risk populations, we may very well wait forever.

## References

[1] Beechar VB, Pouch SM, La Hoz RM. Donor-derived infections: a journey from evidence to policy. Open Forum Infect Dis. 10.1093/ofid/ofae670 PMC11605645 39620107

[2] Boodman C, Garcia OF, Kabbani D, et al Donor-derived *Bartonella quintana* infection in solid organ transplantation: an emerging public health issue with diagnostic challenges. Open Forum Infect Dis 2024; 11:ofae381. 39192995 10.1093/ofid/ofae381 PMC11348938

[3] Kabbani D, Orenbuch-Harroch E, Boodman C, et al Donor-derived bartonellosis in solid organ transplant recipients from unhoused donors in Alberta. Am J Transplant doi:10.1016/j.ajt.2024.09.026. [published online ahead of print 24 September 2024]. 39326850

[4] Wong J. Alberta doctor sounds alarm after 7 patients contract infection from organ transplants: organ donors had been living with homelessness and were infected themselves. Available at: https://www.cbc.ca/news/canada/edmonton/alberta-doctor-sounds-alarm-after-7-patients-contract-infection-from-organ-transplants-1.7364500.

[5] Beeson A, Rich S, Russo M, et al *Bartonella quintana* transmission by kidney transplantation to two recipients from a donor experiencing homelessness, 2022. Emerg Infect Dis 2024; 30: S49–55. 39592261 10.3201/eid3012.240310 PMC11616653

[6] Boodman C, Gupta N, Nelson CA, van Griensven J. *Bartonella quintana* endocarditis: a systematic review of individual cases. Clin Infect Dis 2024; 78:554–61. 37976173 10.1093/cid/ciad706

[7] Boodman C, Gupta N, van Griensven J, Van Bortel W. *Bartonella quintana* detection among arthropods and their hosts: a systematic review and meta-analysis. Parasit Vectors 2024; 17:328. 39095833 10.1186/s13071-024-06413-3 PMC11295871

[8] OPTN . OPTN 10-step policy development process. Available at: https://optn.transplant.hrsa.gov/media/3115/optn-policy-development-process-explanatory-document.pdf. Accessed 26 October 2024.

